# Draft genome sequences of six bacterial isolates from aphid-infested sugar beet and witloof chicory

**DOI:** 10.1128/mra.00343-26

**Published:** 2026-06-25

**Authors:** Nicolas Rojas-Preciado, Wouter Poppelsdorf, Lara Berings, Sara Van Hee, Hans Jacquemyn, Bart Lievens

**Affiliations:** 1 CMPG Laboratory for Process Microbial Ecology and Bioinspirational Management (PME&BIM), Department of Microbial and Molecular Systems (M2S), KU Leuven 26657, Leuven, Belgium; 2 Leuven Plant Institute (LPI), KU Leuven 26657, Leuven, Belgium; 3 Laboratory of Plant Conservation and Population Biology, Biology Department, KU Leuven 26657, Leuven, Belgium; Indiana University Bloomington, Bloomington, Indiana, USA

**Keywords:** aphid-infested plants, draft genome sequences, crop protection

## Abstract

Aphid-infested plants can recruit beneficial bacteria that support plant protection and offer potential for sustainable aphid management. We present the draft genome sequences of six bacteria isolated from aphid-infested sugar beet and witloof chicory. These resources will support the identification of traits relevant to plant-microbe-insect interactions and aphid biocontrol.

## ANNOUNCEMENT

Aphids (Hemiptera: Aphididae) are among the most economically important agricultural pests worldwide (1, 2). Aphid management has relied on synthetic pesticides, but their effectiveness has declined due to widespread resistance development (3). Furthermore, concerns over non-target effects and regulatory constraints have driven a shift toward biological control and integrated pest management (IPM) to reduce chemical inputs while maintaining effective pest suppression (4).

Insect herbivory can promote the recruitment of beneficial bacteria that contribute to plant protection, either directly through insecticidal activity or indirectly by enhancing plant defenses or producing volatiles that attract natural enemies (5–8). Investigating bacteria from aphid-infested plants may, therefore, facilitate the discovery of specific taxa and bioactive traits with potential applications in biological control strategies against aphid pests. Here, we report the draft genome sequences of six plant-associated bacterial isolates from aphid-infested plants. Three strains were isolated from the phyllosphere of sugar beet (*Beta vulgaris* “Lisanna KWS”) infested with the green peach aphid *Myzus persicae* (PME40.26/003, PME40.26/004, and PME40.26/002), and three from witloof chicory roots (*Cichorium intybus* var. *foliosum* “Darling”) infested with the wooly lettuce root aphid *Pemphigus bursarius* (PME37.24/006, PME37.24/025, and PME37.24/033). The isolates comprise one *Kosakonia*, two *Pantoea*, and three *Pseudomonas* strains.

Genomic DNA was extracted from cultures grown in Luria Broth (28°C and 130 rpm) to an OD_600_ of 1, using the DNeasy UltraClean Microbial Kit (Qiagen), according to the manufacturer’s protocol. Illumina 150 bp paired-end libraries were prepared using the NGS DNA Library Prep Set PT004 (Novogene) and sequenced on a NovaSeq X Plus Series (PE150) platform by Novogene (UK). All downstream analyses were performed with default parameters unless otherwise indicated. Raw reads were assessed with FastQC v0.12.1 (9). High-quality paired reads (Phred > 30) were filtered and adapters removed using Trimmomatic v0.40 (10). Contamination check was done with Kraken2 v0.0.2 (11), *de novo* genome assembly with SPAdes v4.2.0 (12) in isolate mode, and contigs shorter than 200 bp were discarded. Assembly completeness and quality were evaluated using QUAST v5.2.0 (13) and CheckM2 v.1.1.0 (14). The average genome coverage of the assemblies based on filtered reads mapped to assemblies was estimated with BBMap v.1.3.0 (15). Species assignment and calculation of the average nucleotide identity (ANI) to the closest reference genome were performed using GTDB-Tk v2.3.2 (16) and AutoMLST2 (17). Kraken2, BBMap, and GTDB-Tk analyses were conducted on the KBase platform (18), CheckM2 was executed using the Galaxy platform (19), and all remaining tools were run in Jupyter notebooks (20). Genome assemblies were annotated with the NCBI Prokaryotic Genome Annotation Pipeline v6.10 (21).

The draft genomes showed an average coverage of 304× and 28 contigs per strain, with largest contigs of 906.51 ± 206.36 kbp and *N*_50_ values ranging from 325.6 to 1,112.7 kbp. Genome size and GC content were higher in *Pseudomonas* isolates (5.67 ± 0.80 Mbp; 60.7 ± 1.7%) than in the other isolates (4.96 ± 0.05 Mbp; 55.4 ± 0.4%) (Table 1).

**TABLE 1:** Summary of genome assembly parameters of six bacterial isolates from the sugar beet phyllosphere infested with the green peach aphid *Myzus persicae*, or from witloof chicory roots infested with the wooly lettuce root aphid *Pemphigus bursarius*

Parameter/strain	PME40.26/002	PME40.26/003	PME40.26/004	PME37.24/006	PME37.24/025	PME37.24/033
Species ID^a^	*Pantoea agglomerans*	*Pseudomonas shirazensis*	*Kosakonia cowanii*	*Pseudomonas* sp.	*Pseudomonas* sp.	*Pantoea agglomerans*
GenBank accession no.	JBVHNO000000000	JBVHNN000000000	JBVHNM000000000	JBVHNL000000000	JBVHNK000000000	JBVHNJ000000000
SRA accession no.	SRR36826332	SRR36826331	SRR36826330	SRR36826329	SRR36826328	SRR36826327
Closest assembly^a^	GCF_001598475.1	GCF_014268785.2	GCF_001975225.1	GCF_020386635.1	GCF_003732275.1	GCF_001598475.1
Average nucleotide identity (%)	98.47^b^	97.38^b^	99.65^b^	94.51^c^	95.21^d^	98.53^b^
Origin	Sugar beet phyllosphere	Sugar beet phyllosphere	Sugar beet phyllosphere	Witloof chicory roots	Witloof chicory roots	Witloof chicory roots
No. of Illumina reads (M)	12.8	11.5	10.1	14.8	11.7	11.4
No. of filtered reads (M)	11.5	10	9	12.8	10.3	10.2
Genome size (Mbp)	5.0	5.27	4.98	5.14	6.59	4.91
Average coverage (×)	348	285	272	373	234	312
No. of contigs	28	19	35	12	38	35
Largest contig (kbp)	726.3	957.2	739.4	1,166.70	722.7	1,126.70
Contig *N*_50_ (kbp)	405.6	843.9	325.6	1,112.70	346.5	355.3
GC content (%)	55.1	62.1	55.9	61.2	58.7	55.2
Genome completeness (%)	100	99.99	100	100	100	100
Genome contamination (%)	0.11	0.01	0.22	0.69	0.69	0.77
No. of CDS	4,910	5,005	4,692	4,927	6,195	4,716
No. of tRNA	59	65	69	55	58	63
No. of rRNA	10	5	6	3	8	12

^*a*^ Taxonomic classification and closest assembly identification were done using GTDB-Tk and AutoMLST2.

^*b*^ Closest assembly match using AutoMLST2 identified as “Type Strain” of the corresponding species.

^*c*^ Taxonomic classification and closest assembly identification based only on closest placement analysis in GTDB-Tk.

^*d*^ Taxonomic classification and closest assembly identification based only on AutoMLST2 (no taxonomic assignment in GTDB-Tk).

The genomes reported may provide a basis for identifying microbial traits relevant to sustainable crop protection and plant-microbe-insect interactions, and expand our knowledge on the bacterial diversity in economically important crops.

## Data Availability

The draft genome assemblies have been submitted to GenBank, and the raw, pre-assembled reads have been deposited in the SRA. The corresponding accession numbers are provided in Table 1. All generated data are associated with BioProject PRJNA1402734, hosted by NCBI.
